# Roles of tissue-resident immune cells in immunotherapy of non-small cell lung cancer

**DOI:** 10.3389/fimmu.2023.1332814

**Published:** 2023-12-07

**Authors:** Rui Tang, Haitao Wang, Mingxi Tang

**Affiliations:** ^1^ School of Basic Medical Sciences, Southwest Medical University, Luzhou, Sichuan, China; ^2^ Department of Pathology, Affiliated Hospital of Southwest Medical University, Luzhou, Sichuan, China; ^3^ The School of Clinical Medical Sciences, Southwest Medical University, Sichuan, Luzhou, China; ^4^ Department of Pathology, Yaan People’s Hospital (Yaan Hospital of West China Hospital of Sichuan University), Yaan, Sichuan, China

**Keywords:** non-small cell lung cancer, tissue-resident immune cells, immunotherapy, tumor immune microenvironment, immune checkpoint inhibitors

## Abstract

Non-small cell lung cancer (NSCLC) is the most common and lethal type of lung cancer, with limited treatment options and poor prognosis. Immunotherapy offers hope for improving the survival and quality of life of NSCLC patients, but its efficacy depends on the tumor immune microenvironment (TME). Tissue-resident immune cells are a subset of immune cells that reside in various tissues and organs, and play an important role in fighting tumors. In NSCLC, tissue-resident immune cells are heterogeneous in their distribution, phenotype, and function, and can either promote or inhibit tumor progression and response to immunotherapy. In this review, we summarize the current understanding on the characteristics, interactions, and roles of tissue-resident immune cells in NSCLC. We also discuss the potential applications of tissue-resident immune cells in NSCLC immunotherapy, including immune checkpoint inhibitors (ICIs), other immunomodulatory agents, and personalized cell-based therapies. We highlight the challenges and opportunities for developing targeted therapies for tissue-resident immune cells and optimizing existing immunotherapeutic approaches for NSCLC patients. We propose that tissue-resident immune cells are a key determinant of NSCLC outcome and immunotherapy response, and warrant further investigation in future research.

## 1 Introduction

Non-small cell lung cancer (NSCLC) represents the majority of lung cancer cases, accounting for approximately 80% to 85% of diagnoses and contributing to a significant number of cancer-related deaths worldwide (1, 2). Despite advancements in surgical techniques, chemotherapy, radiotherapy, and targeted therapy, the prognosis for NSCLC patients remains discouraging with a 5-year survival rate of only 19% (1). Consequently, there is an urgent and imperative need to develop more innovative and effective therapeutic strategies to combat NSCLC.

In recent years, immunotherapy has emerged as a promising approach in the treatment of various cancers, including NSCLC (3, 4). This novel therapeutic technique aims to harness and enhance the body’s natural immune system to recognize and eliminate tumor cells. The efficacy and mechanism of immunotherapy largely depend on the intricate interplay of immune cells, which serve as the main drivers of the immune response. Within this vast array of immune cells, tissue-resident immune cells play a pivotal role in tumor immunotherapy. Distinguished by their long-lasting presence, these immune cells possess memory and effector functions (5). Notable examples of tissue-resident immune cells include tissue-resident memory T (TRM) cells, B cells, macrophages, natural killer (NK) cells, etc. (6). Tissue-resident immune cells can establish immune memories within tissues, enabling them to initiate robust immune responses upon encountering similar or identical antigens. Additionally, tissue-resident immune cells can serve as targets or biomarkers for immunotherapy, imparting both synergistic and antagonistic effects when used in combination with different immunotherapies.

In this review, we address the following question: How do tissue-resident immune cells regulate NSCLC immunity and immunotherapy? We hypothesize that tissue-resident immune cells have a dual role in NSCLC, exerting both anti-tumor and pro-tumor effects depending on their subsets and activation states. We argue that tissue-resident immune cells represent a promising target for improving the efficacy and overcoming the resistance of immunotherapy for NSCLC.

We will first introduce the distribution, phenotype, and function of tissue-resident immune cells in NSCLC tissues. Then, we will discuss the role of tissue-resident immune cells in NSCLC control, including their anti-tumor or pro-tumor effects, and their interactions with other immune or non-immune cells. Next, we will examine the application of tissue-resident immune cells in NSCLC immunotherapy, covering the currently employed immune checkpoint inhibitors (ICIs), along with other state-of-the-art immunotherapeutic approaches and ongoing research advancements. Finally, we will conclude by emphasizing the importance and uniqueness of tissue-resident immune cells in NSCLC immunity and immunotherapy, and by proposing future research directions in this field.

With this comprehensive introduction, it is our intent to emphasize the crucial role and unique status of tissue-resident immune cells in the context of NSCLC. Further studies in this direction will contribute significantly to the development of novel therapeutic strategies, including the targeted modulation of tissue-resident immune cells, exploration of synergistic effects with other therapeutic modalities, and optimization of the application of immune cells in individualized therapy. We firmly believe that by fully harnessing the potential of immunotherapy, we will drastically improve the prognosis of NSCLC patients and pave the way for the future advancements in this field.

## 2 Tissue-resident immune cells in NSCLC

### 2.1 Phenotype and distribution of tissue-resident immune cells in NSCLC tissues

In NSCLC, the phenotypes of tissue-resident immune cells are diverse, including T lymphocytes, macrophages, B lymphocytes, NK cells, dendritic cells (DC), and neutrophils (6). Tumor-associated macrophages (TAM) and tissue-resident memory T cells (TRM) are two important subsets of immune cells that play crucial roles in the tumor immunology in NSCLC.

TAM are derived from circulating monocytes that are recruited to the tumor site by various chemokines and cytokines, such as CCL2, CSF-1, and VEGF (7, 8). Macrophages differentiate differentially in tissues with different micro-environments and can be divided into two distinct polarization states: M1 macrophages (M1) and M2 macrophages (M2). But TAM are generally characterized by M2-like macrophages (9), which exhibit immunosuppressive and protumorigenic functions, such as promoting angiogenesis, lymphangiogenesis, invasion, metastasis, and resistance to therapy (10). TAM are distributed unevenly within the tumor tissue, and their spatial localization can affect their interaction with other immune cells and tumor cells (9). Tissue-resident macrophages gather near tumor cells at an early stage of tumor initiation, enhancing epithelial-mesenchymal transition and the ability of tumor cells to invade (11). Additionally, tissue-resident macrophages trigger a strong response from regulatory T cells, which shields tumor cells from adaptive immunity (11). TAM are potential targets for immunotherapy in NSCLC, and several strategies have been developed to modulate TAM, such as limiting monocytes recruitment, targeting TAM activation, reprogramming TAM into anti-tumor activity, and targeting TAM specific markers (12). However, there are still many challenges and limitations in TAM-targeted therapy, such as the lack of specific and reliable biomarkers, the complexity and diversity of TAM subsets, the dynamic and context-dependent nature of TAM, and the potential adverse effects of TAM manipulation.

TRM is a subset of memory T cells that reside in non-lymphoid tissues, such as the lung, skin, and gut, and provide rapid and robust protection against local infections and tumors (13). TRM differentiation is guided by transcription programs common to both effector and memory T cells (14). CD103 promotes the localization and retention of CD8^+^ T cells in tumor tissues (15) through binding to E-calmodulin (16) and also modulates the CD8^+^ T cell response to anti-tumor immunotherapy (17). CD8+ T cells accumulate in non-small cell lung cancer tumors and accumulate mainly in the tumor center (18). TRM can exert immune pressure on tumor cells at the early stage of tumor development, affecting tumor evolution and escape (19). The number and function of TRM are closely related to tumor prognosis and treatment response (20). TRM is a promising target for NSCLC immunotherapy, for example as a target for improved cancer vaccines, as a target for immune checkpoint blocking, and as a target for adoptive cell therapy (21). However, there are also many challenges and limitations in TRM-targeted therapy, such as the lack of specific and reliable biomarkers, the complexity and diversity of TRM subsets, the dynamic nature of TRM, and the potential adverse effects of TRM manipulation.

In addition to TAM and TRM, other immune cells also play important roles in NSCLC. For example, in early NSCLC tissues, there are large numbers of tissue-resident macrophages and tumor-infiltrating lymphocytes (TILs) (11, 22). The distribution of NK cells in lung tumors is not homogeneous and is concentrated in the central regions of the tumors (18). NK cells are the first line of defense against environmental destruction, pathogens and cancers, enriched in the tumor microenvironment of NSCLC (23), lung trNK cells are prone to produce CCL5, MIP-1β, and GM-CSF, which may always alter their microenvironment by attracting other immune cells (e. g., monocytes, T cells, and eosinophils) (24). A study has shown that tissue-resident CD69^+^ CXCR6^+^ NK cells with depletion phenotype accumulate in human non-small cell lung cancer and are a promising target for immune checkpoint inhibitors in the treatment of NSCLC (25). For DC, a study has shown that DC derived from NSCLC has immunosuppressive effects, and the co-suppressor molecule B7-H3 plays a crucial role in mediating the T cell inhibition of DC under tumor conditions (26). Tissue-resident B cells are a subset of B cells that reside in the tissues and form ectopic lymphoid organs called tertiary lymphoid structures (TLS) in the margins of solid tumors. TLS are associated with improved patient survival and response to immune checkpoint blockade in NSCLC (27). Another study suggests that neoadjuvant chemotherapy may improve the efficacy of ICBs by increasing the density and diversity of tumor-infiltrating lymphocytes, including B cells, as well as inducing immunogenic cell death and antigen release (28). TIL-B is only present in patients with specific tumors, but its increase in number can positively affect clinical outcomes (29). According to *in vitro* assays, TIL-B can be used as local APCs for secondary stimulation of CD4^+^ TILs in NSCLC, ultimately changing its phenotype and function (29). Among the TILs, T cells dominate, while B cells and NK cells also hold importance (22). Tissue resident neutrophils (TRNs) of NSCLC include TANs and NANs (30),NSCLC has immune features dominated by neutrophils (31). TANs are closely involved in tumor proliferation, invasion, and metastasis through the synthesis and secretion of related proteases, such as neutrophil enzymes (NE) and matrix metalloproteinases (MMPs) (32). Salcher et al. demonstrated that tissue-resident neutrophils play a role in NSCLC by participating in TLS formation, acquiring new functional properties in the tumor microenvironment, and expressing ERV antigens. Their presence and function may be modulated by different therapeutic interventions, such as ICB and targeted therapy (30).

In single-cell sequencing, new immune cell subpopulations, such as CD45 AMS, TAN-1, TAN-2, TAN-3, TAN-4, CD45 AMs Can support the proliferation of lung cancer cells *in vivo* (33). TAN-1 shows a high expression of interleukin 1 receptor antagonist (IL 1 RN), which negatively regulates IL-8 secretion to control excessive neutrophil inflammatory activity (34), and the potent NF- κ B activators RIPK271 and CD44, regulating cell recruitment and adhesion (35, 36). The TAN-2 subcluster is characterized by the expression of the major histocompatibility complex (MHC) class II genes HLA-DRA, CD74, HLA-DMB, and HLA-DRB 1, indicating that the phenotype is characterized by immunogenic antigen presentation. The TAN-3 subcluster is characterized by high expression of proinflammatory cytokines (C15orf48, CCL 3, CCL 4, CSTB) and galectin 3 (LGALS3), which is associated with neutrophil activation and migration. TAN-4 shows high expression of ribosomal genes (e. g., RPS 12, RPL 3, RPN 2, RPL 23) and eventually changes to other cellular phenotypes, such as tumor endothelial cells (30).

These results suggest that tissue-resident immune cells have a complex and diverse phenotype and distribution in NSCLC, and they play an important role in the regulation of tumor immune response and immunotherapy.

### 2.2 Interaction of tissue-resident immune cells with the tumor microenvironment

The tumor microenvironment (TME) of NSCLC is a complex and dynamic system comprising various elements, such as cancer cells, immune cells, stroma cells, blood vessels and extracellular matrix (ECM) (37). The TME modulates the behavior and fate of tissue-resident immune cells through multiple mechanisms, influencing the efficacy of the antitumor immunity. In this section, we explore how tissue-resident immune cells interact with the TME and how this affects NSCLC progression and therapy.

First, the migration and settlement of tissue-resident immune cells in the TME are intricately regulated by chemokines and adhesion molecules. Chemokines, such as small molecules like CCL2, CCL7, CXCL248, play a pivotal role in inducing immune cells to move in a specific direction (38). On the other hand, adhesion molecules, such as ICAM-1, VCAM-1, CD103, facilitate immune cell attachment to other cells or ECM (39). In the case of NSCLC, tumor cells and mesenchymal stromal cells exhibit a remarkable ability to secrete diverse chemokines and express various adhesion molecules. These results in the attraction or repulsion of different types of tissue-resident immune cells into or out of tumor tissues. For instance, CCL2 has the capability to recruit M2-type macrophages into tumor tissues (8), thereby inhibiting the effector function and antitumor effects of CD8^+^ T cells (40), which is detrimental to the survival of non-small cell lung cancer patients (41); CD103 promotes the localization and retention of CD8^+^ T cells in tumor tissues (15) through binding to E-calmodulin (16) and also modulates the CD8^+^ T cell response to anti-tumor immunotherapy (17). Hence, it is evident that chemokines and adhesion molecules play crucial regulatory roles in the migration and settlement of tissue-resident immune cells in NSCLC.

Second, the activation and proliferation of tissue-resident immune cells in the tumor microenvironment are tightly regulated by antigen presentation and co-stimulatory signals. Antigen presentation involves immune cells recognizing and binding specific antigens, such as tumor-specific or mutant antigens, through special receptors. This recognition then triggers an immune response, which plays a crucial role in fighting against tumors (42). On the other hand, co-stimulatory signaling refers to the process through which immune cells receive and transmit positive or negative signals via non-specific receptors, thus either enhancing or inhibiting the immune response (43). In NSCLC, several types of antigen-presenting cells (APCs) are present in the tumor microenvironment, such as dendritic cells, macrophages, and B cells (44). These APCs can present antigens to CD8^+^ T cells or CD4^+^ T cells via major histocompatibility complex (MHC) class I or class II molecules, which activate the effector functions of tissue-resident immune cells (42),and these APCs also play a crucial role in regulating the proliferation and survival of tissue-resident immune cells. They achieve this by expressing a wide range of co-stimulatory molecules, such as CD80, CD86, PD-L1, and PD-L2. For example, CD80 and CD86 can bind to CD28, thereby providing positive co-stimulatory signals to promote the proliferation and survival of tissue-resident immune cells (45); PD-L1 and PD-L2 can bind to PD-1(programmed death protein 1), thereby providing negative co-stimulatory signals to inhibit the proliferation and survival of tissue-resident immune cells, promoting NSCLC (46, 47). Thus, antigen presentation and co-stimulatory signaling have important regulatory roles in NSCLC for the activation and proliferation of tissue-resident immune cells.

**Figure 1:**
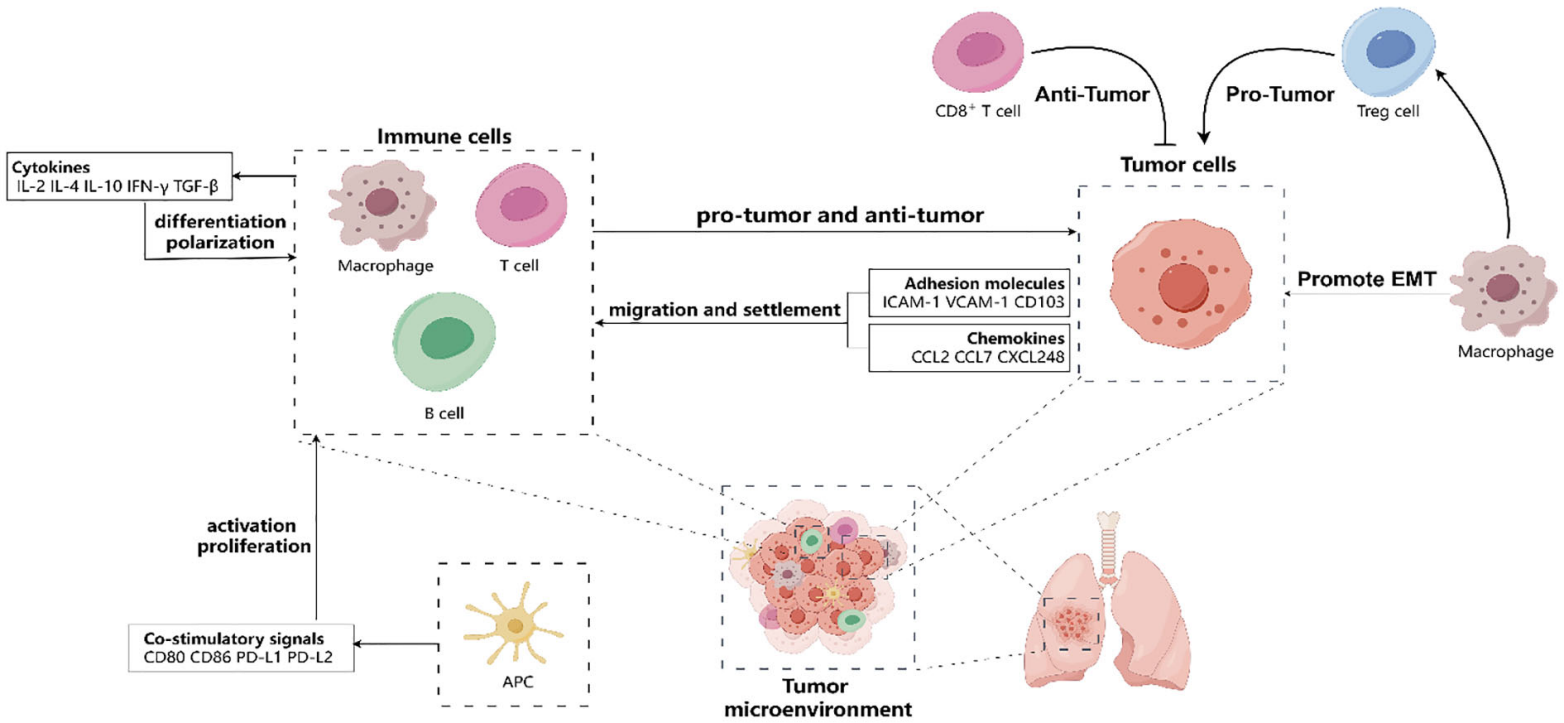
Tumor microenvironment (TME) affect the migration and settlement of tissue-resident immune cells (such as T cells, B cells, macrophages, antigen-presenting cells) by releasing various chemokines (such as CCL2, CCL7, CXCL248, etc.) and expressing various adhesion molecules (such as ICAM-1, VCAM-1, etc.). Besides, tissue-resident immune cells regulate their activation and proliferation by receiving and transmitting co-stimulatory signals (such as CD28/CD80/CD86 or PD-1/PD-L1/PD-L2, etc.) in the TME. Moreover, tissue-resident immune cells regulate their differentiation and polarization by cytokines (such as IL-2, IL-4, IL-10, IFN-γ, TGF-β, etc.) in the TME. In TME, CD8^+^ cells play an anti-tumor role, but macrophages can promote tumor progression directly through epithelial-mesenchymal transformation (EMT) and indirectly through regulating the function of Treg cells. Hence, the TME has a significant impact on the behavior and fate of tissue-resident immune cells, which in turn affects NSCLC progression and therapy.

Third, the differentiation and polarization of tissue-resident immune cells are regulated by cytokines in the tumor microenvironment. Cytokines are proteins that act as messengers between immune cells, which including IL-2, IL-4, IL-10, IFN-γ, and TGF-β (48). The tumor microenvironment of NSCLC contains different types of cytokines that can bind to specific receptors on tissue-resident immune cells and change their phenotype and function. For instance, IL-4 can make CD4^+^ T cells become Th2 cells and help B cells produce antibodies (49); IFN-γ can make CD4^+^ T cells become Th1 cells and boost the effector function of CD8^+^ T cells (50); and TGF-β can make CD4+ T cells become Treg cells and suppress the effector function of CD8^+^ T cells (51). Therefore, cytokines are important regulators of tissue-resident immune cells differentiation and polarization in NSCLC (**Figure 1**).

### 2.3 Identification and characterization of tissue-resident immune cells in NSCLC

Understanding the role and potential of tissue-resident immune cells in NSCLC requires their identification and characterization. However, this task is challenging due to the heterogeneity, diversity, and plasticity of tissue-resident immune cells. Multiple methods and criteria are necessary to identify and characterize tissue-resident immune cells in NSCLC tissues accurately.

One such method is flow cytometry, a technique that can assess the expression of various surface or intracellular markers on individual cells. This allows for quantifying and examining different immune cell subsets and their phenotypes (52, 53). Flow cytometry can also sort the immune cells based on their markers for further analysis (54). Nevertheless, flow cytometry requires predefined antibodies and panels for specific markers, which may limit the discovery of novel or rare immune cell subsets. Furthermore, flow cytometry does not provide information about the spatial distribution and localization of immune cells within NSCLC tissues.

Another method is immunohistochemistry (IHC). IHC utilizes specific antibodies to detect the expression of a small number of markers on tissue sections. Staining or using fluorescence allows for the visualization of immune cells (55). IHC can provide valuable information about immune cells’ spatial distribution and localization and their interactions with tumor cells in the TME. For example, Righi et al. (56) used IHC to classify NSCLC into different subtypes based on the expression of cytokeratin (CK), thyroid transcription factor-1 (TTF-1), and p63. They found that the immunophenotype of NSCLC-not otherwise specified (NOS) was associated with treatment outcome and survival. Specifically, they found that NOS with adenocarcinoma-like (ADC-like) features had a similar survival as ADC. In contrast, NOS with squamous cell carcinoma-like (SCC-like) features had a similar survival as SCC. Moreover, NOS with no differentiation features (“null” group) had a significantly worse survival than the other groups (56). These results suggest that IHC can improve the accuracy of NSCLC typing and guide the optimal treatment strategy. Nevertheless, IHC has low sensitivity and specificity and cannot quantify immune cell subsets’ expression level or frequency. Moreover, it does not offer functional information regarding immune cells in NSCLC tissues. Multiplex immunohistochemistry (mIHC) has been developed to simultaneously detect multiple antigens in a single tissue section using chromogens or fluorophores to overcome these challenges. mIHC can increase the number of parameters that can be measured in a single experiment, reduce tissue consumption, and enable the automated quantification and analysis of the data. For example, Sun et al. (57) used an automated chromogenic mIHC assay to simultaneously detect six biomarkers (CD3, CD8, CD163, PD-L1, FoxP3, and CK) and DAPI in NSCLC. They found that this assay could accurately reveal the spatial distribution and relationship of tumor cells and immune cells in the TME without compromising the antigenicity, cross-reactivity, or staining intensity. They also found that this assay could be performed on standard antigen retrieval and automated staining protocols, reducing the need for validation strategies. Furthermore, mIHC can be enhanced by optimizing the staining protocol, the antibody panel, and the image analysis software. For example, Sun et al. (58) developed an enhanced 7-color mIHC protocol. They used this protocol to analyze the specimens of NSCLC, and found the differences in the composition and distribution of immune cells among different tumor types. These examples demonstrate the advances of mIHC in immunophenotype profiling of NSCLC.

A third method is single-cell sequencing, which can analyze the transcriptome or proteome of individual cells and reveal the gene expression or protein abundance of thousands of genes or proteins. Single-cell sequencing can identify and characterize novel or rare immune cell subsets based on their gene expression or protein abundance profiles, as well as their functional states and pathways (59). Single-cell sequencing can also reveal the heterogeneity and diversity of immune cells in NSCLC tissues (60). Nevertheless, single-cell sequencing is expensive and complex, and requires high-quality samples and bioinformatics tools for data processing and analysis.

Therefore, identifying and characterizing immune cells in NSCLC tissues requires a combination of multiple methods and criteria that can complement each other’s strengths and weaknesses. Using these methods and criteria, researchers can better identify and characterize the tissue-resident immune cells in NSCLC tissues and understand their role in tumor immunity. (All mechanisms in this chapter are summarized in **Table 1**).

**Table 1:** Various mechanisms of tissue-resident immune cells in non-small cell lung cancer.

Interaction Of Tissue-Resident Immune Cells With The Tumor Microenvironment	Complex Regulation Of Chemokines And Adhesion Molecules	CCL2 Can Recruit M2 Type Macrophages To Inhibit The Effector Function And Anti-Tumor Effect Of CD8^+^ T Cells And Promote The Development Of NSCLC.
		CD103, By Binding To E-Calmodulin, Promotes The Localization And Retention Of CD8^+^ T Cells In Tumor Tissue And In Response To Anti-Tumor Immunotherapy.
	Strict Regulation Of Antigen Presentation And Co-Stimulatory Signals	CD80, CD86 Bind To CD28 To Promote The Proliferation And Survival Of Tissue-Resident Immune Cells And Inhibit NSCLC.
		PD-L1, PD-L2 Can Bind To PD-1 To Inhibit The Proliferation And Survival Of Tissue-Resident Immune Cells And Promote NSCLC.
	The Regulation Of The Cytokines	IL-4 Enables The Conversion Of CD4^+^ T Cells Into Th 2 Cells And Helps B Cells To Produce Antibodies.
		IFN- Γ Can Transform CD4^+^ T Cells Into Th 1 Cells And Enhance The Effector Function Of CD8^+^ T Cells
		TGF- β Can Make CD4^+^ T Cells Become Treg Cells And Inhibit The Effector Function Of CD8^+^ T Cells.
Role Of Tissue-Resident Immune Cells In The Control Of Non-Small-Cell Lung Cancer	Anti-Tumor Effect	TRM Cells Recognize Tumor Antigens, Mediate Cytotoxicity And Cytokine Secretion, And Also Enhance The Function Of Other Immune Cells. It Can Also Upregulate PD-1 Expression And Produce IFN- Γ.
		The Trnk Cells Can Recognize And Kill Tumor Cells Through Mechanisms Such As ADCC, Ncrs And NKG2D, And They Can Also Produce Cytokines To Regulate TME And Promote The Activation Of Other Immune Cells.
		Tissue Resident B Cells Form Tertiary Lymphoid Structures (TLS), Which Are Associated With Improved Survival And Response To Immune Checkpoint Blockade In NSCLC Patients. Til-B Can Act As A Local Apc To Secondary Stimulate CD4+ Til In Non-Small Cell Lung Cancer, Ultimately Altering Its Phenotype And Function.
	Synergy Or Antagonistic Effects With Other Immune Or Non-Immune Cells	Lung Trnk Cells Are Prone To Produce CCL5, MIP-1β, And GM-CSF, Which May Always Alter Their Microenvironment By Attracting Other Immune Cells (E. G., Monocytes, T Cells, And Eosinophils)
		CD103^+^ TRM Produces GZMB And IFN-Γ To Enhance The Recruitment Of Monocytes, NK Cells, And XCR1^+^ Cdc1 To The Tumor Sites To Fight The Tumor.
		The Reduction Of Tissue-Resident Macrophages Reduced The Number Of Treg Cells And Altered The Phenotype. Promote Accumulation Of CD8^+^ T Cells And Inhibit Tumor Development.
	Tumor Promotion And Injury Promotion Effects	Tissue-Resident Immune Cells Can Induce Inflammation By Secreted Cytokines, Chemokines, And Growth Factors, And Multiple Proinflammatory Mediators Participate In Complex Inflammatory Signaling To Promote Tumor Progression And Metastasis.
		Tissue-Resident Macrophages Enhance Epithelial-Mesenchymal Transition And The Ability Of Tumor Cells To Invade, And Trigger A Strong Response From Regulatory T Cells, Which Shields Tumor Cells From Adaptive Immunity.
		PD-1 And CTLA-4 Blockade Partially Improved Their Immune Failure.
		The Co-Inhibitory Molecule B7-H3 Mediates The T-Cell Inhibition Of DC In Tumor Conditions.

## 3 The role of tissue-resident immune cells in the control of non-small cell lung cancer

### 3.1 Antitumor effects of tissue-resident immune cells

Tissue-resident immune cells can exert anti-tumor effects by directly killing tumor cells, producing cytokines and chemokines, activating and recruiting other immune cells, and influencing the TME. Several studies have shown that tissue-resident immune cells are associated with better clinical outcomes and response to immunotherapy in NSCLC patients. For example, tissue-resident memory T (TRM) cells are characterized by the expression of CD69 and CD103 (61). These cells have been found to be enriched in non-small cell lung cancer tumors and are associated with improved survival and reduced recurrence rates (20, 62, 63). TRM cells can recognize tumor antigens and mediate cytotoxicity and cytokine secretion (62, 64). Moreover, TRM cells can enhance the function of other immune cells, such as dendritic cells and NK cells, through direct contact or soluble factors (65). TRM cells can also respond to immunotherapy by upregulating PD-1 expression and producing IFN-γ (20, 66).

Another example of tissue-resident immune cells with anti-tumor activity is tissue-resident natural killer (trNK) cells, which are a subset of innate lymphoid cells that express CD69 and CD49a (24). trNK cells are abundant in the lung and can recognize and kill tumor cells through various mechanisms, such as antibody-dependent cellular cytotoxicity (ADCC), natural cytotoxicity receptors (NCRs) and NKG2D (67–69). trNK cells can also produce cytokines, such as IFN-γ, TNF-α, and IL-15, that modulate the TME and promote the activation of other immune cells (24, 25).

Subcentral clusters in the TRN of NSCLC include TANs and NANs (30), and TAN abundance in the TME was considered as the primary predictor of adverse outcome (70). A high neutrophil to CD8 T-cell ratio within the tumor is a poor prognostic indicator of relapse-free survival and OS (71). The antitumor activity of TANs includes activation of the expression of immune cytokines, low levels of arginase, high H2O2 production, and increased ability to kill tumor cells *in vitro* (72). At the same time, it can also directly produce cytotoxic mediators or release TNF-related apoptosis-inducing ligand to inhibit tumor growth (73). In resected lung cancer tissues, TANs crosstalk with activated T cells, resulting in a significant upregulation of co-stimulatory molecules on the surface of neutrophils, thereby promoting the proliferation of T cells in the positive feedback cycle (74).

Other types of tissue-resident immune cells, such as tissue-resident macrophages, tissue-resident B cells, and non-classical T cells (such as γδ T cells) (75), may also contribute to anti-tumor immunity in NSCLC by various mechanisms. However, the exact anti-tumor role and function of these tissue-resident immune cells in NSCLC remain to be elucidated.

### 3.2 Synergistic or antagonistic effects of tissue-resident immune cells with other immune or non-immune cells

Tissue-resident immune cells in non-small cell lung cancer do not act in isolation, but rather interact with other immune cells in the tumor microenvironment. These interactions can have either synergistic or antagonistic effects on the anti-tumor immunity and the tumor progression. For example, in human, CD103^+^ TRM cells can produce granzyme B (GZMB) and IFN-γ, which can enhance recruitment of monocytes, NK cells, and XCR1^+^ cDC1 to the tumor site, resulting in anti-tumor (76, 77). Nevertheless, several dysfunctional subtypes of TRM cells exist in human NSCLC tissues, including NKG2A^+^CD8^+^ T cells, NKG2A is an inhibitor of both T cells and NK cells. Persistent activation thereof may cause T cells and NK cells to express NKG2A and potentially lead to chronic infection or cancer (78). In NSCLC, MDSC, expressing Arg-1 and iNOS, participates in inhibiting CD8^+^ T cell proliferation and reducing CD3 ζ expression (79, 80). MDSCs also release various immunosuppressive molecules and upregulate repression checkpoints to promote differentiation or development of Tregs and interfere with homing and trafficking of tumor-specific T cells (81). Myeloid DC plays a central role in antitumor immunity because of their ability to initiate T cells in lymph nodes. DC can also produce TGF- β, which can induce the differentiation of CD4^+^ T lymphocytes to Treg, thereby inhibiting T cell proliferation (82). CD103 DC controls CD8^+^ T cell activation in a variety of tumor types (83).

On the other hand, tissue-resident macrophages accumulate around tumor cells early during tumorigenesis to promote epithelial-mesenchymal transition and tumor cell invasiveness, as well as to elicit a potent regulatory T-cell response protecting tumor cells from adaptive immunity. Depletion of tissue-resident macrophages reduced the number and altered the phenotype of Treg cells. This led to the accumulation of CD8+ T cells and reduced tumor invasiveness and growth (11).

### 3.3 Tumor-promoting and injury-promoting effects of tissue-resident immune cells

Tissue-resident immune cells play a complex role in cancer, as they can exert both anti-tumor and pro-tumor effects. Tissue-resident immune cells can promote tumor initiation, progression, and metastasis by various mechanisms, such as inducing inflammation and epithelial-mesenchymal transition (EMT). Moreover, tissue-resident immune cells can also compromise the anti-tumor immunity or cause tissue damage, for instance through immune exhaustion.

Inflammation is a key factor that links chronic infection, tissue injury, and cancer. Tissue-resident immune cells can induce inflammation by secreting cytokines, chemokines, growth factors, which can recruit and activate other immune cells or non-immune cells in the TME. Whereas activated immune cells and inflammatory cells produce reactive oxygen species (ROS) and reactive nitrogen species (RNS), which exert chemical effects in inflammation-driven carcinogenesis (84). And ROS-induced DNA damage includes DNA strand breaks, DNA base modifications, and DNA cross-links, leading to replication errors and genomic instability, which in turn lead to tumorigenesis (85). Moreover, in the tumor microenvironment, multiple pro-inflammatory mediators are involved in complex inflammatory signaling, which promotes tumor progression by promoting tumor cell extravasation through the stroma (86). Inflammation can also promote tumor metastasis by enhancing tumor cell intravasation, extravasation, and colonization at distant sites (87).

EMT is the process of acquiring mesenchymal features and losing epithelial features by epithelial cells (88). Using lineage tracing, it has been shown that the development of NSCLC lesions in mice initially accumulates TRMs in the vicinity of the tumor cells to promote epithelial-mesenchymal transition and invasiveness in the tumor cells, and that these TRMs also induced potent Treg cell responses that protected the tumor cells from adaptive immune responses (11, 89). In summary, EMT can promote tumor progression by enhancing tumor cell invasion, migration and resistance to apoptosis or therapy. EMT can also promote tumor metastasis by facilitating tumor cell detachment from the primary site and attachment to the secondary site (90, 91).

Immune exhaustion is the process of losing effector functions and becoming unresponsive to stimulation. Some tissue-resident immune cells, especially CD8^+^ T cells, can become exhausted after chronic exposure to tumor antigens or immunosuppressive signals. This exhaustion can also be identified by means of CD38 labeling (92). And, it has been found that a tissue-resident CD69 CXCR6 NK cell with an exhaustive phenotype accumulates in NSCLC. Its dysfunction can be partially ameliorated by PD-1 and CTLA-4 blockade (25). Thus, these findings suggest a close interrelationship between tissue-resident immune cells exhaustion and tumor. Understanding and intervening in this interrelationship through research could help develop more effective tumor treatments.

## 4 The application of tissue-resident immune cells in non-small cell lung cancer immunotherapy

### 4.1 The role, limitations, and clinical studies of immune checkpoint inhibitors

Immune checkpoint inhibitors (ICIs) are a type of immunotherapy that block the negative signals between tumor cells and immune cells, thereby enhancing the anti-tumor immune response (93). ICIs targeting PD-1/PD-L1 or CTLA-4 have shown remarkable efficacy and survival benefit in patients with advanced NSCLC, especially those with high PD-L1 expression or tumor mutational burden (94, 95). However, ICIs are not effective for a subset of patients, and some patients may develop primary or secondary resistance, or experience immune-related adverse events (96, 97). Therefore, it is important to understand the mechanisms of resistance and toxicity, and to identify biomarkers that can predict the response and outcome of ICIs.

One of the factors that may influence the efficacy of ICIs is the presence and function of tissue-resident immune cells in the TME. Tissue-resident immune cells can provide local immune surveillance and protection against pathogens and tumors, but they can also be modulated by the TME to acquire pro-tumoral or immunosuppressive phenotypes (5). Therefore, tissue-resident immune cells may play a dual role in NSCLC immunotherapy, either enhancing or inhibiting the anti-tumor effect of ICIs.

Several studies have investigated the impact of tissue-resident immune cells on the response and resistance to ICIs in NSCLC. For example, a recent study by Zhang et al. (19) showed that tissue-resident memory T (TRM) cells exerted early immune pressure on NSCLC cells and shaped their clonal evolution and neoantigen landscape. The authors found that TRM cells were enriched in tumors with high PD-L1 expression and low tumor mutational burden, and that they recognized shared neoantigens that were present in most tumor cells (19). However, TRM also induced the emergence of subclones that escaped TRM recognition by losing or mutating the neoantigens (19). These subclones were resistant to PD-1 blockade and had poor prognosis. The authors suggested that targeting TRM-induced neoantigens or combining PD-1 blockade with other therapies that prevent tumor evolution may improve the outcome of NSCLC immunotherapy (19).

Another study by Lee et al. (98) investigates the presence and expansion of TRM in the peripheral blood of patients with lung cancer who are undergoing immune checkpoint inhibitor therapy. This study reveals that treatment with ICIs leads to the clonal expansion of TRM in the peripheral blood, suggesting that these cells play a crucial role in the anti-tumor immune response induced by ICIs.

These studies reveal a pivotal role of tissue-resident immune cells in modulating the TME and the response to ICIs in NSCLC, highlighting the potential of these cells as therapeutic targets. However, more studies are needed to elucidate the diversity, plasticity and function of tissue-resident immune cells in different types and stages of NSCLC, and to explore how to manipulate them to enhance the efficacy and safety of ICIs.

### 4.2 Other immunotherapeutic approaches and research progress

Besides ICIs, other immunotherapeutic approaches have been explored for NSCLC, such as vaccines, adoptive cell therapy, and oncolytic viruses. These strategies aim to enhance the anti-tumor activity of tissue-resident immune cells or recruit more tissue-resident immune cells to the tumor site.

Vaccines are designed to elicit specific immune responses against tumor-associated antigens (TAAs) or neoantigens (99, 100). Several types of vaccines have been tested in NSCLC, including peptide-based, dendritic cell-based, tumor cell-based, and viral vector-based vaccines (101–104). Some of these vaccines have shown promising results in combination with ICIs or chemotherapy (100, 105). However, the efficacy of vaccines is limited by the heterogeneity of TAAs, the low immunogenicity of neoantigens, and the immunosuppressive TME. Therefore, more efforts are needed to identify optimal antigens, delivery systems, and combination regimens for NSCLC vaccines.

Adoptive cell therapy (ACT) involves the infusion of ex vivo expanded or genetically modified immune cells into patients. The most widely used ACT for NSCLC is chimeric antigen receptor (CAR) T cell therapy, which targets specific antigens expressed by tumor cells or stroma (106, 107). CAR T cells have shown remarkable efficacy in hematological malignancies (108, 109), but their application in solid tumors is challenging due to the lack of tumor-specific antigens, the poor infiltration and persistence of CAR T cells in the TME, and the hostile TME that inhibits CAR T cell function (110). Several strategies have been proposed to overcome these obstacles, such as engineering CAR T cells with enhanced migratory and survival abilities, co-expressing cytokines or checkpoint modulators, and combining CAR T cells with other immunotherapies (111, 112).

Oncolytic viruses (OVs) are genetically modified or naturally occurring viruses that selectively infect and lyse tumor cells, while stimulating innate and adaptive immune responses. OVs can also be used as vectors to deliver therapeutic genes, such as cytokines, co-stimulatory molecules, or checkpoint inhibitors (113, 114). Several OVs have been evaluated in NSCLC preclinical and clinical studies, such as Coxsackievirus A21 (V937), Coxsackievirus A11, influenza viruses and Vesicular stomatitis virus (VSV) (115–117). A study that tested the safety and effectiveness of a modified version of CVA11, called V937, in patients with advanced solid tumors, including NSCLC. V937 was given alone or with another drug called pembrolizumab, which blocks a molecule called PD-1 that can weaken the immune system. The combination of V937 and pembrolizumab showed some signs of shrinking the tumors (117).

Bispecific antibodies are a promising strategy that targets both PD-L1 and TGF-β, two key immunosuppressive factors in the tumor microenvironment. TGF-β is a pleiotropic cytokine that can inhibit the activation, proliferation, and cytotoxicity of T cells (118, 119), as well as induce the differentiation of Treg cells and myeloid-derived suppressor cells (119). Moreover, TGF-β can promote the epithelial-mesenchymal transition of tumor cells, which increases their invasiveness and resistance to ICIs (120). By blocking both PD-L1 and TGF-β, bispecific antibodies can synergistically enhance the antitumor immune response and overcome the resistance to PD-L1 blockade (121). Several anti-TGF-β/PD-L1 bispecific antibodies have been developed and tested in preclinical and clinical studies, such as YM101, BiTP, and M7824. YM101 is a bispecific antibody that targets murine PD-L1 and TGF-β, and has shown potent antitumor activity in mouse models of triple-negative breast cancer (122). BiTP is a humanized version of YM101 that targets human PD-L1 and TGF-β, and has demonstrated superior antitumor efficacy to anti-PD-L1 and anti-TGF-β monotherapy in humanized mouse models of TNBC (122). M7824 is a bifunctional fusion protein that consists of a PD-L1 antibody and the extracellular domain of TGF-β receptor II, and has entered phase I/II trials for various solid tumors, including NSCLC (123). These bispecific antibodies have the potential to improve the clinical outcomes of NSCLC patients who are refractory or resistant to PD-L1 blockade.

Other immunotherapeutic approaches that are under investigation for NSCLC include cytokine therapy, innate immune stimulators, and microbiome modulation (124–126). Some examples of other immunotherapeutic approaches related to NSCLC are LunX-CART, CD147-CART, and OSE2101 vaccine. LunX-CART is a CAR T cell therapy that targets LunX, a molecular marker of overexpression in NSCLC (106). LunX-CART has been shown to effectively eradicate LUNX-positive NSCLC cells by secreting cytokines (106). Cd147-cart is another CAR-T cell therapy targeting CD147. CD147, a glycoprotein overexpressed in non-small cell lung cancer, plays an important role in NSCLC progression and is a promising target for CART therapy in NSCLC (127). CD147-CART has demonstrated substantial antitumor activity and safety in preclinical models of non-small cell lung cancer (127). OSE2101 is a peptide-based vaccine that contains modified epitopes from five tumor-associated antigens restricted to HLA-A2^+^ patients (128). OSE2101 has been evaluated in a randomized controlled trial of NSCLC patients who failed immune checkpoint inhibitors. The results showed that OSE2101 improved overall survival compared to standard care in patients with secondary resistance to immune checkpoint inhibitors (128). These examples illustrate how the immune system can be reignited to fight against tumors by targeting specific antigens, enhancing CAR T cell function, or inducing neoantigen-specific responses. These approaches aim to enhance the activation and recruitment of tissue-resident immune cells or modulate the TME to favor anti-tumor immunity. However, the optimal combination and sequencing of these therapies with ICIs or conventional treatments remain to be determined.

### 4.3 Challenges and issues of immunotherapy for NSCLC

Although immunotherapy for NSCLC has made remarkable achievements, there are still many challenges and issues that need to be addressed. These include the identification of reliable biomarkers, the management of immune-related adverse events (irAEs), the overcoming of primary and acquired resistance, and the optimization of combination and sequential therapies.

Biomarkers are essential for selecting suitable patients, predicting treatment response, monitoring disease progression, and evaluating treatment efficacy. The most widely used biomarker for NSCLC immunotherapy is PD-L1 expression, which is measured by immunohistochemistry (IHC) on tumor biopsy samples (129). However, PD-L1 expression is not a perfect predictor of response. First, only patients with both high PD-L1 expression and high immune infiltration could benefit from chemotherapy plus immunotherapy in first-line treatment of advanced NSCLC (130). Moreover, PD-L1 expression is dynamic and heterogeneous, and may vary depending on the tumor site, stage, histology, and prior treatments. Therefore, there is an urgent need to identify more robust and comprehensive biomarkers for NSCLC immunotherapy. Some potential candidates include tumor mutational burden (TMB), microsatellite instability (MSI), (131), gene expression profiles (GEPs) and microbiome (131–135). TMB is defined as the total number of somatic/acquired mutations per coding area of a tumor genome (Mut/Mb). It reflects the neogenic antigen load of the tumor and correlates with the response to immunotherapy (136). MSI refers to microsatellite instability caused by the accumulation of DNA replication errors due to the loss or mutation of mismatch repair genes, leading to abnormal expression of proteins in tumor cells and activation of the immune system (137). GEPs refer to gene expression profiles, which can reflect the molecular characteristics and status of tumor cells and immune cells, as well as the immune phenomena in the TME (138).

The performance of these biomarkers and their limitations still require further validation and optimization. For example, the measurement methods, standards, and thresholds of TMB have not been unified, and different detection platforms and algorithms may lead to different results (139). There are also some issues with the detection of MSI, such as the selection of microsatellite loci, the sensitivity and specificity of detection techniques, and the dynamic changes of MSI. More clinical studies on the relationship between MSI-related sites and tumor resistance are needed to improve the therapeutic effect of chemotherapy (140). The application of GEPs is also limited by factors such as sample quality and quantity, data analysis and interpretation, and cost considerations.

In order to improve the predictive ability of biomarkers, it is necessary to integrate these different biomarkers and construct more precise and personalized models. Some studies have already attempted this approach, such as combining TMB and PD-L1, which has been found to be more predictive of the response to immunotherapy in NSCLC patients than individual biomarkers (141). In addition, some studies have used machine learning and artificial intelligence methods to extract the most valuable features from a large amount of biomarker data and establish more complex and refined prediction models (142).

The application of these biomarkers also needs to consider their clinical feasibility and practicality. The detection of some biomarkers requires sufficient tumor tissue samples, which is a barrier for patients who cannot undergo or have difficulty with biopsies. Therefore, it is necessary to develop non-invasive or minimally invasive detection methods, such as using circulating tumor DNA (ctDNA) or circulating tumor cells (CTCs) in blood or other fluids, to predict the clinical response of anti-PD-1/PD-L1 therapy in cancer (143). On the other hand, the detection of some biomarkers requires the use of high-throughput sequencing or chip technologies, which are costly and require specialized equipment and personnel, limiting their use in resource-limited areas. Therefore, it is necessary to develop more convenient and economical detection methods, such as using multiplex PCR or multiplex IHC to detect specific genes or proteins (57, 144).

The research on these biomarkers also needs to explore their future directions and prospects. Some biomarker studies are still in their early stages and require more clinical trials and cohort studies to validate their effectiveness and reliability. Some biomarker studies also need to consider their interaction and impact with other treatment modalities, such as radiotherapy, chemotherapy, targeted therapy, etc., as well as the influence of different treatment regimens and sequences on biomarkers. Some biomarker studies also need to focus on the differences and applicability of biomarkers in different tumor subtypes and molecular subtypes, as well as in different populations and regions. Some biomarker studies also need to seek more new and potential biomarkers, as well as new and potential detection methods and technologies.

In conclusion, biomarkers are an important area in tumor immunotherapy, which requires more research and innovation to discover and develop more effective biomarkers. Future research should focus on improving the detection and predictive capabilities of biomarkers, integrating different biomarkers to construct more precise and personalized models, considering the clinical feasibility and practicality of biomarkers, and exploring new directions and prospects for biomarkers.

irAEs are a unique spectrum of side effects caused by immunotherapy, which result from the activation of immune cells against normal tissues (145). irAEs can affect various organs and systems, such as skin, gastrointestinal tract, liver, endocrine glands, lungs, kidneys, nervous system, and cardiovascular system (146, 147). The incidence and severity of irAEs vary depending on the type and combination of ICIs, the tumor type and stage, and the patient characteristics (148). The management of irAEs usually involves the use of corticosteroids or other immunosuppressive agents, which may compromise the anti-tumor efficacy of ICIs (149, 150). Therefore, it is important to monitor and diagnose irAEs early, and to develop strategies to prevent or treat them without affecting the anti-tumor response.

Resistance to immunotherapy is a major obstacle for achieving durable clinical benefits in NSCLC patients. Resistance can be classified into primary resistance, which occurs when tumors do not respond to ICIs at all, and acquired resistance, which occurs when tumors initially respond to ICIs but then relapse or progress (151). The mechanisms of resistance are complex and multifactorial, involving both intrinsic factors of tumor cells and extrinsic factors of TME. Some of the intrinsic factors include genetic alterations in tumor antigens (152), antigen presentation machinery (153), interferon signaling pathway (154), chromatin remodeling (155), and other inhibitory immune checkpoints (156). Some of the extrinsic factors include immunosuppressive cells (such as Treg cells, myeloid-derived suppressor cells, tumor-associated macrophages) (157, 158), soluble mediators (such as cytokines, chemokines, metabolites) (159) and vascular factors (such as hypoxia) (160). To overcome resistance to immunotherapy, several approaches have been proposed or tested in preclinical or clinical studies. These include identifying novel immune checkpoints or targets, modulating TME to enhance immune infiltration or activation, combining ICIs with other immunotherapies or conventional therapies (such as chemotherapy, radiotherapy, targeted therapy), and developing personalized or neoantigen-based vaccines.

Combination and sequential therapies are promising strategies to improve the efficacy and overcome the resistance of ICIs for NSCLC. However, there are many challenges and issues that need to be addressed before these strategies can be widely applied in clinical practice. These include the selection of optimal agents or modalities to combine with ICIs, the determination of optimal doses and schedules of each component, the evaluation of potential synergistic or antagonistic effects between different therapies, the identification of predictive biomarkers for each combination or sequence, the assessment of cost-effectiveness and quality of life for each combination or sequence, and the management of increased toxicity or irAEs caused by each combination or sequence.

## 5 Conclusion

In this review, we have discussed the role of tissue-resident immune cells in non-small cell lung cancer (NSCLC) immunity and immunotherapy. Tissue-resident immune cells are a heterogeneous population of immune cells that reside in various tissues, including the lung. Tissue-resident immune cells have distinct phenotypes, functions, and interactions with the tumor microenvironment (TME) compared to their circulating counterparts. Tissue-resident immune cells can exert anti-tumor or pro-tumor effects depending on their subsets and activation states. Tissue-resident immune cells are also involved in the response and resistance to immunotherapy, especially immune checkpoint inhibitors (ICIs), which are the mainstay of treatment for advanced NSCLC.

Tissue-resident immune cells represent a promising target for improving the efficacy and overcoming the resistance of ICIs for NSCLC. Several strategies have been proposed or tested to modulate tissue-resident immune cells in NSCLC, such as enhancing their recruitment, activation, or persistence in the TME, or inhibiting their immunosuppressive or tumor-promoting functions. Moreover, tissue-resident immune cells can be combined with other immunotherapeutic approaches, such as vaccines, adoptive cell therapy, oncolytic viruses, or bispecific antibodies, to achieve synergistic anti-tumor effects. However, there are still many challenges and issues that need to be addressed before tissue-resident immune cells can be widely applied in clinical practice. These include the identification of reliable biomarkers for tissue-resident immune cells, the optimization of delivery systems and dosing regimens for tissue-resident immune cells-based therapies, the evaluation of potential toxicities or adverse events caused by tissue-resident immune cells manipulation, and the development of personalized or precision medicine based on tissue-resident immune cells.

In conclusion, tissue-resident immune cells play an important role and have a special status in NSCLC immunity and immunotherapy. Future research directions should focus on developing novel tissue-resident immune cells-targeted drugs, exploring the synergistic effects of tissue-resident immune cells with other treatment modalities, and optimizing the application of tissue-resident immune cells in individualized therapy.

## Author contributions

RT: Writing – original draft. HW: Writing – original draft. MT: Funding acquisition, Writing – review & editing.
